# Signal beyond nutrient, fructose, exuded by an arbuscular mycorrhizal fungus triggers phytate mineralization by a phosphate solubilizing bacterium

**DOI:** 10.1038/s41396-018-0171-4

**Published:** 2018-06-13

**Authors:** Lin Zhang, Gu Feng, Stéphane Declerck

**Affiliations:** 1 College of Resources and Environmental Sciences; Research Center for Resources, the Environment and Food Safety, China Agricultural University, 100193 Beijing, China; 2 Université catholique de Louvain, Earth and Life Institute, Applied Microbiology, Mycology, Croix du sud 2, bte L7.05.06, 1348 Louvain-la-Neuve, Belgium

## Abstract

Cooperation is a prevalent phenomenon in nature and how it originates and maintains is a fundamental question in ecology. Many efforts have been made to understand cooperation between individuals in the same species, while the mechanisms enabling cooperation between different species are less understood. Here, we investigated under strict in vitro culture conditions if the exchange of carbon and phosphorus is pivotal to the cooperation between the arbuscular mycorrhizal fungus (AMF) *Rhizophagus irregularis* and the phosphate solubilizing bacterium (PSB) *Rahnella aquatilis*. We observed that fructose exuded by the AMF stimulated the expression of phosphatase genes in the bacterium as well as the rate of phosphatase release into the growth medium by regulating its protein secretory system. The phosphatase activity was subsequently increased, promoting the mineralization of organic phosphorus (i.e., phytate) into inorganic phosphorus, stimulating simultaneously the processes involved in phosphorus uptake by the AMF. Our results demonstrated for the first time that fructose not only is a carbon source, but also plays a role as a signal molecule triggering bacteria-mediated organic phosphorus mineralization processes. These results highlighted the molecular mechanisms by which the hyphal exudates play a role in maintaining the cooperation between AMF and bacteria.

## Introduction

How species adapt to their environment to survive and reproduce is a central question in ecology. Some organisms favor selfish acts in order to maintain a competitive advantage [1], while others engage in a wide range of cooperative behaviors from cooperation between genes to cooperation between communities [2]. Yet, cooperation remains the least known, and several theories, e.g. kin selection, reciprocity…, have been explored by scientists [3].

The association between plants and arbuscular mycorrhizal fungi (AMF), that goes back more than 450 million years [4], represents a suitable model for studying the cooperation between unrelated species [5, 6]. AMF produce substantial hyphae biomass in soil (between 45 and 100 m mycelium per gram of soil in prairies and pastures [7]) and recruit various genera of bacteria on its surface [8, 9]. For instance, phosphate solubilizing bacteria (PSB) involved in the mineralization of organic phosphates have been detected on the AMF hyphae surface [10, 11]. Via their metabolism, inorganic P is released and exchanged for plant-derived carbohydrates exuded by hyphae [12–14]. If it is unanimously accepted that hyphae exudates provide nutrients to the microbes developing in their close vicinity [15], then the mechanisms by which exudates may stimulate phosphorus mineralization by bacteria are still unknown, as well as the potential candidate signaling compounds.

The pivotal role of sugars as signaling molecules in microorganisms has been widely illustrated. For example, UDP-glucose is a potential intracellular signal molecule in the control of expression of sigma S and sigma S-dependent genes in *Escherichia coli* [16]. Fructose metabolism has been reported to affect cellular processes such as biofilm formation by streptococci and bacterial pathogenicity in plants [17]. Trehalose is able to regulate the bacterial community composition [13].

Interestingly, in the symbiotic association between plants and AMF, it has been shown that the plants release sucrose into the peri-arbuscular space, which is then hydrolyzed to fructose and glucose. Both hexoses are probably transferred inside the arbuscules, though this needs to be demonstrated firmly [18]. In parallel, it has been reported that the intraradical hyphae of AMF can take up exogenous glucose and fructose directly [19–21]. Both are major sugars of root exudates [22]. Part of the hexoses is then synthetized to glycogen in the intraradical hyphae and further transported to the extraradical hyphae where is it converted into trehalose [18]. However, it cannot be ruled out that part of the glucose and fructose is not converted and remains under these forms in the extraradical hyphae. Trehalose, glucose, and fructose may thus be important sugars inside the extraradical hyphae which could be potentially released outside the hyphae. Interestingly, these three sugars were reported in hyphal exudates of *Rhizophagus irregularis* MUCL 41833 [23], even though the authors could not firmly exclude that their presence was due to diffusion from the roots. Furthermore, no information was available on the concentration of these sugars in the hyphosphere and whether they could represent candidate signaling compound that might trigger the C–P exchange process between AMF and PSB.

In the present study, we investigated whether fructose, glucose, and trehalose are present in the hyphal exudates of *R. irregularis* MUCL 43194 and if they are potential candidate signaling compounds to prime the processes related to metabolism of cell and phosphatase production in the PSB *Rahnella aquatilis* HX2. More specifically, we addressed the following hypotheses: (1) AMF hyphal exudates contain sugars, which are preferentially assimilated by the bacteria, (2) the expression of genes encoding bacterial phosphatase production and excretion increase concomitantly with the uptake of specific sugars, (3) the genes involved in the processes of phosphate transfer and polyP synthesis in the extraradical hyphae of the AMF are enhanced by the presence of the bacteria.

## Materials and methods

### Biological material

The AMF strain used was *R. irregularis* (Błaszk., Wubet, Renker & Buscot) C. Walker & A. Schüßler as “*irregulare*” MUCL 43194. The fungus was maintained in root organ cultures (ROC) of carrot (*Daucus carota* L.). The PSB strain used was *R. aquatilis* strain HX2. This strain has been whole-genome sequenced [24] and was shown effective in the mineralization and utilization of phytate P [15]. Two plants were used: ROC of *Daucus carota* L. clone DC2 and whole plants of *Medicago truncatula* L. Gaertn. cv. Jemalong A17 (SARDI, Australia) prepared as in Buysens et al. [25].

### Gene analysis

The whole-genome sequence of *R. aquatilis* is available at the National Center for Biotechnology Information (NCBI) (GenBank assembly accession: GCA_000255535). Three putative sugar (i.e., fructose, glucose, and trehalose) transporters of *R. aquatilis* were selected. More than ten phosphatases were found in *R. aquatilis*, but only the excretive ones can play a role in organic P hydrolyzation in the soil. Because the excretive proteins have a short terminal signal peptide and transmembrane domains, SignalP 4.1 software (http://www.cbs.dtu.dk/services/SignalP) and TMHMM V2.0c program (http://www.cbs.dtu.dk/services/TMHMM/) were used to predict the excretive phosphatases. Finally, one phytase, three acid phosphatases, and one alkaline phosphatase, as well as two key proteins in cell division of *R. aquatilis* and two proteins in type II and IV secretory system, were selected. The primers for the genes of the above selected proteins or their subunits were designed by Primer 5. The sequences of primers and other information related to the selected genes are reported in Table S1.

### Experiment 1: fructose, glucose, and trehalose exudation by hyphae of *R. irregularis*

Bi-compartmented Petri plates (90 × 15 mm) were used to grow the excised transformed carrot roots and AMF as detailed in St-Arnaud et al. [26] (see Methods in supplementary information) with minor modification: 5 ml MSR medium was removed with a sterilized scalpel along the plastic barrier in the root compartment (RC) to avoid diffusion of sugars from RC to hyphal compartment (HC) (see Fig. S1). Three treatments were considered: absence of mycorrhizal roots in the RC and *R. irregularis* in the HC (RC^−MR^/HC^−RI^); presence of mycorrhizal roots in the RC but without proliferation of *R. irregularis* in the HC (RC^+MR^/HC^−RI^); presence of mycorrhizal roots in the RC and proliferation of *R. irregularis* in the HC (RC^+MR^/HC^+RI^). Each treatment consisted of four replicates.

At week 7, the extraradical mycelium (ERM) of AMF in the RC^+MR^/HC^+RI^ treatment developed extensively on the slope of the HC. Ten milliliters of liquid MSR medium without Ca(NO_3_)_2_·4H_2_O, sucrose, and vitamins but containing 280 μM phytate-P (Na-phytate, Sigma-Aldrich, St Louis, USA) was added in the HC to allow the growth of ERM. The ERM extended from the slope into the whole HC. The same liquid MSR medium was used in the RC^−MR^/HC^−RI^ and RC^−MR^/HC^−RI^ treatments.

After another 4 weeks, the HC was covered by actively growing hyphae. The MSR medium was then collected, passed through an Acrodisc^®^ Syringe Filter and stored at −20 °C until analysis. The hyphae remaining in the HC were cleaned three times with sterilized deionized water, collected and weighed, and the content was extracted as described by Duan et al. [27] (see Methods in supplementary information). The presence and concentration of fructose, glucose, and trehalose in the hyphal extract and the collected medium was determined by their respective standard sample (J&K Scientific Ltd., Beijing, China) using ICS-3000 Ion Chromatography System (Dionex, California, USA) [28]. For the analysis of sugars in the hyphal exudates, the RC^−MR^/HC^−RI^ treatment was used as the control, the RC^+MR^/HC^−RI^ treatment to test whether there is an influence of volatile compounds produced by roots and mycorrhizal hyphae, and the RC^+MR^/HC^+RI^ treatment to collect the hyphal exudates.

### Experiment 2: Impact of *R. irregularis* on metabolism and phosphatase production in *R. aquatilis*

Three treatments (RC^−MR^/HC^−RI^, RC^+MR^/HC^−RI^, and RC^+MR^/HC^+RI^) with four replicates were set up as in experiment 1, but without removing 5 mm MSR medium in the RC. At week 11, the HC was covered by the ERM. The remaining liquid MSR medium (±9 ml) was transferred to a 15-ml tube and adjusted to 10 ml using the same liquid MSR medium as above but without phytate-P. Two milliliters of bacteria at a concentration of approximately 10^8^ CFUs ml^−1^ was added and mixed uniformly. The 12 ml MSR medium was then added to the HC of each experimental system in contact with the ERM. The supernatant of bacteria was also added in the RC^−MR^/HC^−RI^ and RC^−MR^/HC^−RI^ treatments following the same procedure (see Fig. S2).

At 1, 6, 12, 24, 48, and 72 h, 0.5 ml medium was sampled and added to 1 ml RNAprotect^®^ Bacteria Reagent (Qiagen) before storage at −80 °C for RNA extraction. At final harvest (i.e., 72 h), the remaining medium (±9 ml) in the HC was passed through an Acrodisc^®^ Syringe Filter (0.2 μm Supor^®^ Membrane, Pall Corporation, New York, USA), to remove the bacterial cells, and stored at −20 °C for analysis of inorganic and total P. Inorganic P concentration was determined by the molybdenum blue method [29]. Total P concentration was evaluated by inductively coupled plasma atomic emission spectroscopy (ICP-AES). Phytate-P concentration was then calculated by subtracting the inorganic P from the total P concentration. Determination of acid and alkaline phosphatase activity (pKatal ml^−1^ medium) was conducted according to Neumann [30].

Total RNA was extracted from the frozen bacterial cells using the RNeasy^®^ Mini Kit (Qiagen), according to the manufacturer’s instructions and treated with TURBO DNA-*free*^TM^ Kit (Ambion) to remove the possible DNA contamination. For single-strand cDNA synthesis, 300 ng of total RNA was reverse-transcribed at 65 °C for 10 min, 55 °C for 20 min, and 85 °C for 5 min in a final volume of 20 μl containing 1 μl random primer using Transcriptor High Fidelity cDNA Synthesis Kit (Roche). The products were then diluted to 100 μl.

Quantitative RT-PCR (qRT-PCR) was performed using a LightCycler® 96 Real-Time PCR System (Roche). Each PCR reaction was carried out in a total volume of 10 μl containing 2.5 μl cDNA, 5 μl 2× FastStart Essential DNA Green Master (Roche), and 0.5 μl of each primer (5 μM). The following PCR program was used: 95 °C for 600 s, 45 cycles of 95 °C for 10 s, 60 °C for 10 s, 72 °C for 10 s. A melting curve was recorded at the end of each run to exclude the generation of non-specific PCR products [31]. All reactions were performed on three technical replicates. Baseline range and threshold cycle (Ct) values were calculated using LightCycler® 96 software. The ΔCt was calculated by subtracting the Ct value of a reference gene from the Ct value of each target gene. Relative change fold of each target gene was normalized by 2^−ΔΔCt^ method, referring to the ΔCt value in the RC^−MR^/HC^−RI^ treatment harvested at the 1-h sampling time. The constitutively expressed RNA polymerase sigma factor RpoD was used as a house-keeping gene.

### Experiment 3: Relationship between phosphatase gene expression and uptake of fructose and glucose in *R. aquatilis*

Ten milliliters of liquid MSR medium (without Ca(NO_3_)_2_·4H_2_O, sucrose, and vitamins) without fructose (0 µM) or supplemented with 5, 20, 100 μM, and 1 mM of fructose and with 20 μM glucose was mixed with 2 ml bacterial suspension of *R. aquatilis* (optical density, OD_600_ = 0.8). The mixed solution was poured into one of the compartments of the bi-compartmented Petri plates and cultured at 27 °C. After 1 and 6 h, the bacterial cells were harvested and the total RNA was extracted. The expression of putative fructose transporter gene *fruT* and phosphatase genes *acp1* and *alp* were determined. The preparation of bacterial suspension, RNA extraction, genes expression determination, and analyses were as described in experiment 2.

### Experiment 4: Influence of fructose and glucose on growth and phosphatase activities of *R. aquatilis*

Similar to experiment 3, *R. aquatilis* was cultured in the absence of fructose or in the presence of 5, 20, 100 μM, and 1 mM of fructose and glucose. At 6, 12, 24, 48, and 72 h, 0.5 ml medium was sampled and the OD was measured at 590 nm using an iMark^TM^ Microplate Reader (Bio-Rad, Hercules, CA, USA). At 7 d, the remaining medium was passed through an Acrodisc^®^ Syringe Filter to remove the bacterial cells and determine the acid and alkaline phosphatase activities.

### Experiment 5: Influence of *R. aquatilis* on gene expression in the extraradical hyphae of *R. irregularis*

An autotrophic in vitro culture system was used (adapted from Voets et al. [32]) (see Methods in supplementary information). Starting from week 3 after transfer of the plantlets, 10 ml MSR medium, lacking sucrose and vitamins, and cooled to 40 °C, was added weekly to the RC. At week 9, when a profuse ERM was growing on the slope, 10 ml liquid MSR medium without Ca(NO_3_)_2_·4H_2_O, sucrose, and vitamins, but containing 280 μM phytate-P, was added to the HC. During the following 4 weeks, 3 ml of the same liquid MSR medium, but without phytate-P, was added twice a week.

At week 13, a profuse ERM covered the HC. Three systems with AMF-colonized plantlets and three control systems with non-mycorrhizal plantlets were selected. The liquid MSR medium in the HC was transferred to a 15-ml tube and adjusted to 10 ml using the same liquid MSR medium as above, but without phytate-P. Two milliliters of bacteria at a concentration ±10^8^ CFUs ml^−1^ was added and mixed uniformly. The 12 ml MSR medium containing the bacteria was added to the HC of each system. Three additional systems with AMF-colonized or non-colonized plantlets were similarly selected and 2 ml liquid MSR medium without *R. aquatilis* was added to the HC. Four treatments were thus considered: absence of *R. irregularis* and *R. aquatilis* in the HC (–RI–RA); absence of *R. irregularis*, but with *R. aquatilis* in the HC (–RI+RA); presence of *R. irregularis* but without *R. aquatilis* in the HC (+RI–RA); presence of both *R. irregularis* and *R. aquatilis* in the HC (+RI+RA). Three replicates were considered per treatment (see Fig. S3).

The liquid MSR medium containing *R. aquatilis* was added in the HC in contact with the ERM. After 6 h, 0.5 ml medium was sampled and added to 1 ml RNAprotect^®^ Bacteria Reagent (Qiagen) according to the handbook to stabilize the bacterial RNA. The samples were then stored at −80 °C until RNA extraction. The expressions of sugar transporter and phosphatase genes were determined. The preparation of bacterial suspension, RNA extraction, gene expression determination, and analyses were as described in experiment 2.

The ERM was collected, poured into a 2-ml tube and stored at −80 °C. The frozen fungal material was ground using MagNA Lyser (Roche) and the total RNA extracted using the RNeasy^®^ Plant Mini Kit (Qiagen), according to the manufacturer’s instructions and treated with TURBO DNA-*free*^TM^ Kit (Ambion) to remove the possible DNA contamination. For single-strand cDNA synthesis, 150 ng of total RNA was reverse-transcribed at 65 °C for 10 min, 55 °C for 20 min, and 85 °C for 5 min in a final volume of 20 μl containing 1 μl Oligo (dT) primer using Transcriptor High Fidelity cDNA Synthesis Kit (Roche). The products were then diluted to 60 μl. The qRT-PCR was performed to determine the phosphate transporter gene *GintPT* and polyP synthesis gene *Vtc4p* [33]. Transcript levels were normalized to the Ct value of *GintEF1α* [34]. The program was similar to experiment 2.

The remaining medium in the HC was passed through an Acrodisc^®^ Syringe Filter to remove the bacterial cells and stored at −20 °C for analysis of inorganic P concentration by the molybdenum blue method.

### Data analysis

Statistical analyses were performed using SPSS v. 16.0 (SPSS Inc., Chicago, IL, USA). In experiment 1, significant differences (*P* < 0.05) among three sugar concentrations in the hyphal exudates were evaluated by a Tukey’s honest significant difference (HSD) test. In experiment 2, one-way ANOVA was conducted to compare the effect of AMF on acid and alkaline phosphatase activities, inorganic P concentration, and phytate-P concentration. Significant differences (*P* < 0.05) among three inoculation treatments were evaluated by a Tukey’s HSD test. Two-way ANOVA was conducted to compare the effects of harvest time (Time), AMF, and their interaction on expression of putative sugar transporter genes *fruT* and *gluT*; phosphatase genes *phy*, *acp1*, *acp2*, *acp3*, and *alp*; key genes in cell division *ftsA* and *ftsZ*, and genes *gspF* and *vib8* involved in type II and IV secretory system. In experiments 3 and 4, significant differences (*P* < 0.05) among the treatments were evaluated by a Tukey’s HSD test. In experiment 5, significant differences in gene expression between two inoculation treatments were evaluated by a *t*-test.

## Results

### Experiment 1: Fructose, glucose, and trehalose exudation by hyphae of *R. irregularis*

Fructose, glucose, and trehalose were detected both inside the extraradical hyphae developing in the HC and in the hyphal exudates. In hyphae, their concentrations were, respectively, 75.6 ± 32.8, 263.9 ± 106.7, and 188.6 ± 84.5 μmol g^−1^ fresh weight, while in exudates they were 20.1 ± 1.9, 15.7 ± 0.7, and 0.17 ± 0.07 μM, respectively.

### Experiment 2: Impact of *R. irregularis* on metabolism and phosphatase production in *R. aquatilis*

**Fig. 1:**
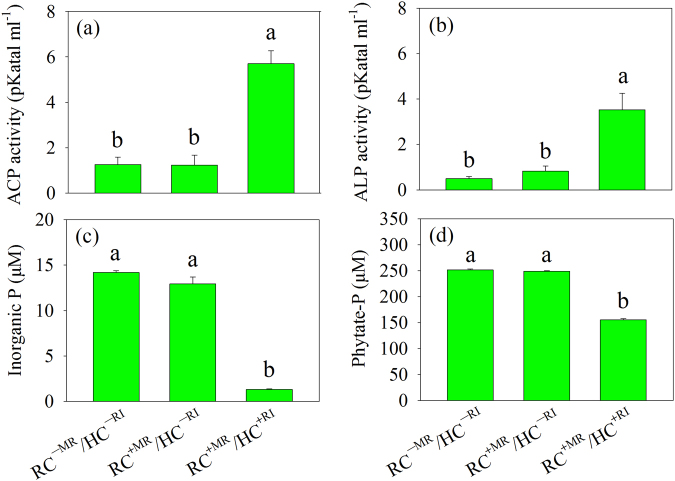
**a** Acid phosphatase (ACP) activity, **b** alkaline phosphatase (ALP) activity, **c** inorganic P concentration, and **d** phytate-P concentration remaining in the liquid MSR medium harvested from the HC of the Petri plate in experiment 2. Histograms with the same letter do not differ significantly (*P* ≥ 0.05; Tukey’s HSD test; *n* = 4). RC^−MR^/HC^−RI^, absence of mycorrhizal roots in the RC and *R. irregularis* in the HC; RC^+MR^/HC^−RI^, presence of mycorrhizal roots in the RC but without proliferation of *R. irregularis* in the HC; RC^+MR^/HC^+RI^, presence of mycorrhizal roots in the RC and proliferation of *R. irregularis* in the HC. RC root compartment, HC hyphal compartment, MR mycorrhizal roots, RI *R. irregularis*

The phosphatase activities and P concentrations in the MSR medium of the HC were measured after 72 h of culture of *R. aquatilis* in the absence or presence of the extraradical hyphae of *R. irregularis* (ANOVA results are shown in Table S2a). No significant differences were noticed in phosphatase activities (Fig. 1a, b) and P concentrations (Fig. 1c, d) between the two control treatments (RC^−MR^/HC^−RI^ and RC^+MR^/HC^−RI^). Acid phosphatase and alkaline phosphatase activities were significantly larger (*P* < 0.05) in the presence of extraradical hyphae in the HC (i.e., RC^+MR^/HC^+RI^ treatment) (Fig. 1a, b) and the inorganic P and phytate-P concentrations significantly smaller (*P* < 0.05) as compared to the two control treatments (Fig. 1c, d).

**Fig. 2:**
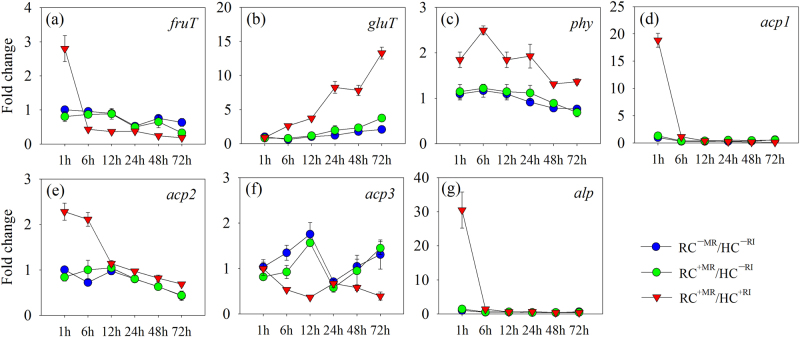
Expression of **a**, **b** putative sugar transporter genes (*fruT* and *gluT*) and **c**–**g** phosphatase genes (*phy*, *acp1*, *acp2*, *acp3*, and *alp*) of *R. aquatilis* harvested from the HC of the Petri plates at different time points in experiment 2. Data are means of four replicates. RC^−MR^/HC^−RI^, absence of mycorrhizal roots in the RC and *R. irregularis* in the HC; RC^+MR^/HC^−RI^, presence of mycorrhizal roots in the RC but without proliferation of *R. irregularis* in the HC; RC^+MR^/HC^+RI^, presence of mycorrhizal roots in the RC and proliferation of *R. irregularis* in the HC. RC root compartment, HC hyphal compartment, RA *R. aquatilis*, MR mycorrhizal roots, RI *R. irregularis*

The expression of putative sugar transporter genes was measured in the *R. aquatilis* grown in the HC in the absence or presence of the extraradical hyphae (i.e., RC^+MR^/HC^+RI^ treatment) (ANOVA results are shown in Table S2b). The expression of putative trehalose transporter gene *treT* was not detected in any treatment, while the other two putative sugar transporter genes (i.e., putative fructose transporter gene *fruT* and putative glucose transporter gene *gluT*) were detected in all treatments and at all observation times (Fig. 2a, b). No significant differences were noticed in gene expression between the two controls, except at 72 h for *fruT* and *gluT*. In the presence of the extraradical hyphae in the HC (i.e., RC^+MR^/HC^+RI^ treatment), the expression of *fruT* was the greatest at 1 h, was significantly smaller (*P* < 0.05) at 6 h, and the following times and did not differ significantly between 6, 12, 24, 48, and 72 h (Fig. 2a). The expression of *gluT* was the smallest at 1 h and then gradually increased until 72 h (Fig. 2b). Compared with the controls, the expression of *fruT* in the RC^+MR^/HC^+RI^ treatment was significantly greater (*P* < 0.05) at 1 h but smaller at 6, 12, 24, 48, and 72 h; the expression of *gluT* in the RC^+MR^/HC^+RI^ treatment was only significantly greater (*P* < 0.05) at 6, 12, 24, 48, and 72 h and did not differ from the controls at 1 h.

The expression of five phosphatase genes expressing exuded enzymes (i.e., *phy*, *acp1*, *acp2*, *acp3,* and *alp*) was measured to study the phosphatase production in *R. aquatilis* (ANOVA results are shown in Table S2b). Besides the expression of *acp1* at 24 h and *acp3* at 6 h, no significant difference was noticed in the expression of genes between the two control treatments. Whatever the harvest time, the expression of *phy* was significantly greater (*P* < 0.05) in the RC^+MR^/HC^+RI^ treatment as compared to the control treatments (Fig. 2c), while the expression of *acp1*, *acp2*, and *alp* was significantly greater (*P* < 0.05) in the presence of the AMF only at 1 and 6 h (Fig. 2d, e, g). The expression of *acp3* was significantly smaller (*P* < 0.05) in the RC^+MR^/HC^+RI^ treatment as compared to the control treatments at 6, 12, 48, and 72 h (Fig. 2f).

**Fig. 3:**
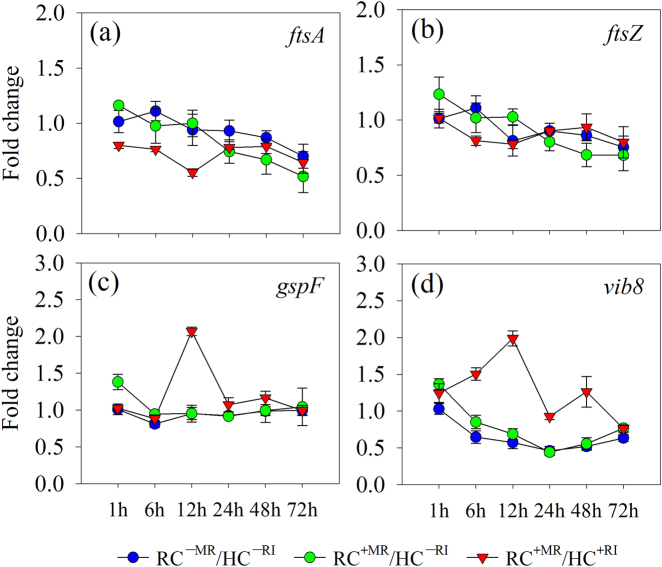
Expression of **a**, **b** key genes in cell division (*fstA* and *fstZ*) and **c**, **d** type II (*gspF*) and type IV (*vib8*) secretory system of *R. aquatilis* harvested from the HC of the Petri plates at different time points in experiment 2. Data are mean of four replicates. RC^−MR^/HC^−RI^, absence of mycorrhizal roots in the RC and *R. aquatilis* in the HC; RC^+MR^/HC^−RI^, presence of mycorrhizal roots in the RC but without proliferation of *R. aquatilis* in the HC; RC^+MR^/HC^+RI^, presence of mycorrhizal roots in the RC and proliferation of *R. aquatilis* in the HC. RC root compartment, HC hyphal compartment, RA *R. aquatilis*, RI *R. irregularis*

The expression of two key genes involved in cell division (*fstA* and *fstZ*) was measured to study the effects of *R. irregularis* on the metabolism of *R. aquatilis* (ANOVA results are shown in Table S2b). The presence of extraradical hyphae in the HC (i.e., the RC^+MR^/HC^+RI^ treatment) significantly inhibited the expression of *fstA* at 1, 6, and 12 h (Fig. 3a) but did not influence *fstZ* (Fig. 3b) as compared to the control treatments.

The expression of two genes involved in type II (*gspF*) and type IV (*vib8*) secretory system was measured to study the effects of *R. irregularis* on phosphatase secretion of *R. aquatilis* (ANOVA results are shown in Table S2b). The presence of extraradical hyphae in the HC (i.e., the RC^+MR^/HC^+RI^ treatment) stimulated the expression of *gspF* at 12 h (Fig. 3c) and of *vib8* at 6, 12, 24, and 48 h as compared to the control treatments (Fig. 3d).

### Experiment 3: Relationship between phosphatase gene expression and uptake of fructose and glucose in *R. aquatilis*

**Fig. 4:**
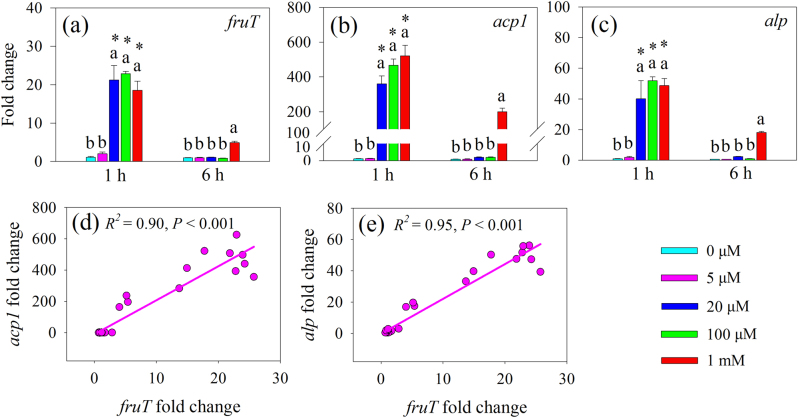
Expression of **a** putative fructose transporter gene (*fruT*) and **b**, **c** phosphatase genes (*acp1* and *alp*) of *R. aquatilis* growing in the liquid MSR medium containing 5, 20, 100 μM, or 1 mM fructose or without (0 μM) this sugar harvested at 1 and 6 h in experiment 3. The associations between the expression of *fruT* and **d** *acp1* (*R*^2^ = 0.90; *P* < 0.001) and **e** *alp* (*R*^2^ = 0.95; *P* < 0.001) of *R. aquatilis* were analyzed. Histograms with the same letter do not differ significantly (*P* ≥ 0.05; Tukey’s HSD test; *n* = 3) among five concentrations of fructose at the same harvest time point and asterisks indicate significant (*P* < 0.05; *t*-test) differences between two harvest time points at the same fructose concentration

Because putative trehalose transporter gene *treT* was not detected, we restricted our observations to the impact of fructose and glucose on phosphatase production in *R. aquatilis*, and to the relationship between the expression of these two putative sugars transporter gene (*fruT* and *gluT*) and phosphatase genes (*acp1* and *alp*). Firstly, the expression of *fruT* was estimated 1 and 6 h after growth of *R. aquatilis* in liquid MSR medium in the absence (0 µM) or in presence of 5, 20, 100 μM, or 1 mM fructose added to the medium. At 1 h, the expression of *fruT* was significantly greater (*P* < 0.05) in the presence of 20, 100 μM, and 1 mM fructose as compared to the presence of 0 and 5 μM fructose, while at 6 h, significant difference was only noticed in the presence of 1 mM fructose as compared to the other concentrations (Fig. 4a). The expression of *acp1* and *alp* showed the same patterns compared to the expression of *fruT* in response to different concentrations of fructose added to the MSR medium (Fig. 4b, c). In addition, the linear regression analysis showed a significant positive relation between the expression of *fruT* and *acp1* (Fig. 4d) or *alp* (Fig. 4e).

**Fig. 5:**
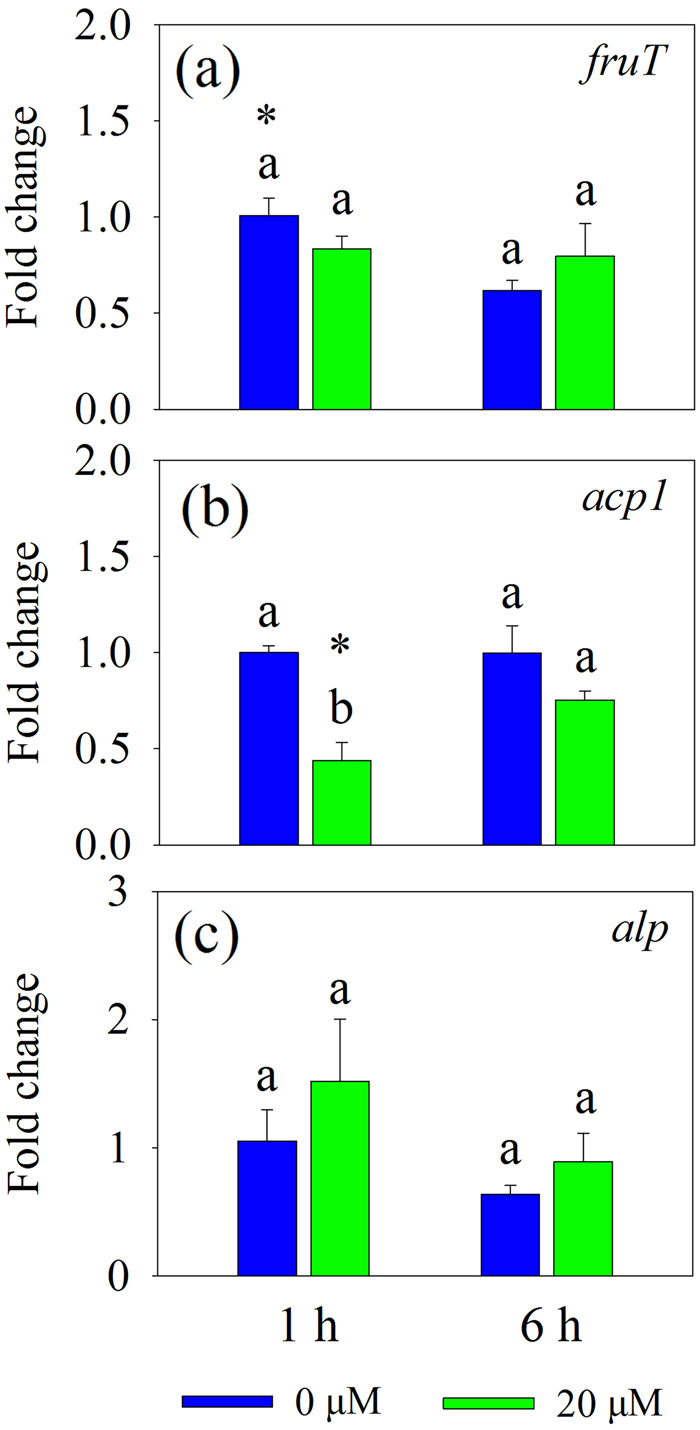
Expression of **a** putative fructose transporter gene (*fruT*) and **b**, **c** phosphatase genes (*acp1* and *alp*) of *R. aquatilis* growing in the liquid MSR medium containing 20 μM glucose or without (0 μM) this sugar harvested at 1 and 6 h in experiment 3. Histograms with the same letter do not differ significantly (*P* ≥ 0.05; *t*-test) among two sugar treatments at the same harvest time point and asterisks indicate significant (*P* < 0.05; *t*-test) differences between two harvest time points at the same sugar treatment

Secondly, the influence of glucose at 20 μM on the phosphatase production in *R. aquatilis* was tested. Glucose at 20 μM had no impact on the expression of *fruT* at 1 and 6 h (Fig. 5a). Similarly, 20 μM glucose did not significantly increase the expression of *acp1* (Fig. 5b) and *alp* (Fig. 5c) as compared to the treatment without (0 µM) glucose.

### Experiment 4: Influence of fructose and glucose on growth and phosphatase activities of *R. aquatilis*

**Fig. 6:**
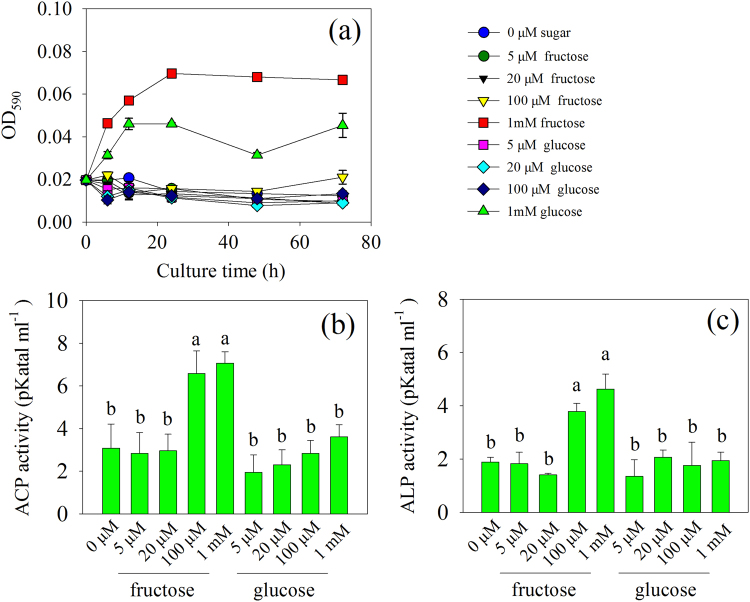
**a** Bacterial growth at 0, 6, 12, 24, 48, and 72 h and **b**, **c** phosphatase activities in the medium at 7 days when *R. aquatilis* was grown in the liquid MSR medium without fructose or glucose (i.e., 0 µM) or containing 5, 20, 100 μM, or 1 mM of the sugars in experiment 4. Histograms with the same letter do not differ significantly (*P* ≥ 0.05; Tukey’s HSD test; *n* = 3) among the treatments

The bacterial growth increased from 0 to 24 h or 0 to 12 h in the presence of 1 mM fructose and glucose, respectively, and then remained unchanged. Fructose at 1 mM had a stronger effect on stimulating bacterial growth than glucose at the same concentration (Fig. 6a). At 7 days, the presence of fructose at 100 μM and 1 mM significantly increased (*P* < 0.05) both the acid and alkaline phosphatase activities, while this was not the case in presence of glucose (Fig. 6b, c).

### Experiment 5: Influence of *R. aquatilis* on genes expression in the extraradical hyphae of *R. irregularis*

**Fig. 7:**
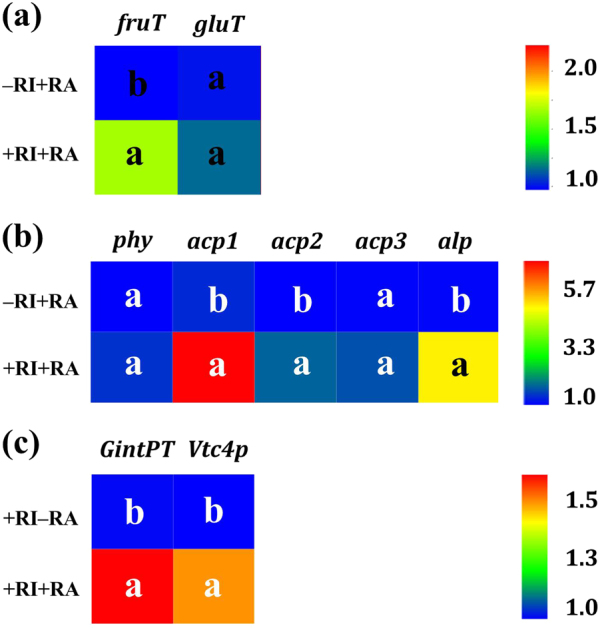
Expression of **a** putative sugar transporter genes (*fruT* and *gluT*) and **b** phosphatase genes (*phy*, *acp1*, *acp2*, *acp3*, and *alp*) of *R. aquatilis*, and **c** phosphate transporter gene *GintPT* and polyP synthesis gene *Vtc4p* of the extraradical hyphae of *R. irregularis* harvested from the hyphal compartment of the Petri plate at 6 h in experiment 5. Different letters indicate significant difference (*P* ≥ 0.05; *t*-test; *n* = 3) between the treatments of –RI+RA and +RI+RA or +RI–RA and +RI+RA. –RI+RA, absence of *R. irregularis* but with *R. aquatilis* in the HC; +RI–RA, presence of *R. irregularis* but without *R. aquatilis* in the HC; +RI+RA, presence of both *R. irregularis* and *R. aquatilis* in the HC. HC hyphal compartment, RA *R. aquatilis,* RI *R. irregularis*

The proliferation of extraradical hyphae in the HC (i.e., the +RI+RA treatment) of the plant in vitro culture system influenced the expression of putative sugar transporters and phosphatase genes of *R. aquatilis* harvested 6 h after plating the bacteria in the extraradical hyphae, as compared to the absence of *R. irregularis* (i.e., the –RI+RA treatment). In the presence of extraradical hyphae, the expression of *fruT* was significantly upregulated by 0.6 fold (*P* < 0.05) and no difference was noticed in the expression of *gluT* as compared to the –RI+RA treatment (Fig. 7a). The expression of *acp1*, *acp2*, and *alp* was significantly upregulated by 4.2, 0.8, and 4.0 fold (*P* < 0.05), respectively, in the presence of extraradical hyphae in the HC (Fig. 7b). In this plant, in vitro culture experiment, the increased expression of *fruT* was also accompanied by a significantly increased expression of *acp1* and *alp*.

In addition, the presence of *R. aquatilis* in the HC (i.e., the +RI+RA treatment) stimulated the expression of the phosphate transporter gene *GintPT* and polyP synthesis gene *Vtc4p* in the extraradical hyphae of *R. irregularis* as compared to the absence of *R. aquatilis* (i.e., the +RI–RA treatment): the expression of *GintPT* and *Vtc4p* was significantly upregulated by 0.7 and 0.5 fold (*P* < 0.05), respectively (Fig. 7c).

## Discussion

During evolution with plants, AMF have lost the genes encoding proteins involved in saprotrophic function [35], which means that they cannot directly breakdown soil organic matter [11, 36]. For instance, AMF cannot utilize phytate-P directly since they are unable to secrete phytases [35]. Part of the inorganic P available to the AMF is released from the enzymatic activities of microbes which are able to decompose organic matter [11]. Microbes may thrive in the close vicinity of AMF forming communities on the surface of the extraradical hyphae. They intimately cooperate with AMF by providing inorganic nutrients released from the decomposition of organic matter in exchange for C exuded by the hyphae [15]. The mechanisms by which these exudates trigger P mineralization process in bacteria and the potential candidates that act as eliciting compounds are still unknown. In the present study, we hypothesized that sugars released by AMF hyphae are not only nutrients for the bacterium [15] but also signal molecules to trigger P mineralization by the bacterium, thus indirectly promoting the acquisition of the mineralized P by the hyphae of the fungus.

To test this hypothesis, we first determined the ability of the extraradical hyphae to exude fructose, glucose, and trehalose. These sugars were detected inside the extraradical hyphae and as exudates, which confirmed the result of Bharadwaj et al. [23]. Trehalose was detected at a larger concentration than fructose but at a smaller concentration than glucose inside the hyphae (i.e., 75.6 ± 32.8, 263.9 ± 106.7, and 188.6 ± 84.5 μmol g^−1^ fresh weight for fructose, glucose, and trehalose, respectively), while its concentration in the exudates was the smallest as compared to fructose and glucose (i.e., 20.1 ± 1.9, 15.7 ± 0.7, and 0.17 ± 0.07 μM for fructose, glucose, and trehalose, respectively). To exclude the possibility that the sugars were released directly from the roots in the RC and diffused inside the medium to the hyphae and then by capillarity from RC to HC via the hyphae, 5 mm medium was removed in the RC along the plastic barrier. A mechanism of diffusion via the sole medium from the RC to HC was thus totally excluded. Transport of sugars via the surface of hyphae via capillary movement could also most probably been discounted also, because the hyphae (in the 5 mm medium gap) developed in the air and water potential on their surface should be drastically reduced, decreasing any possibility for capillary movement. If the transport of sugars via the surface of the hyphae can thus not be formally excluded, it is obvious that the main transport, in our experimental conditions, has taken place through the lumen of the hyphae. Finally, it has been shown that the extraradical hyphae of AMF are unable to take up exogenous glucose and fructose [19–21], therefore excluding the possible uptake by the extraradical hyphae of hexoses released by the roots in the medium and their direct transport inside the hyphae. Collectively, our results demonstrate that fructose, glucose, and trehalose are exuded by the AMF hyphae.

The molecular response of the PSB *R. aquatilis* to the exudation of fructose, glucose, and trehalose was then investigated. As suggested by gene expression results, only fructose and glucose were transferred into *R. aquatilis* within the 72 h of experiment. The absence of detectable gene expression of putative trehalose transporter was curious because *R. aquatilis* is able to use this sugar [37]. One possible explanation is that trehalose may not be the true substrate of the studied gene-encoded protein and other putative sugar transporters may have this role. Another possible explanation is that the bacterium shows a preference for the absorption of the other two sugars over the 72-h study period. This was corroborated by the gene expression analysis indicating a preference of *R. aquatilis* for fructose first and then glucose. In addition, when *R. aquatilis* was cultured with fructose or glucose at the same concentration (i.e., 1 mM) during 72 h, the optical density (OD_590_) increased faster in the presence of fructose than in the presence of glucose. It supported our first hypothesis that sugars in the hyphal exudates are preferentially assimilated in sequential order by the bacterium. This mechanism was observed previously and has been named C catabolite repression [38]. Most bacteria can selectively use substrates from a mixture of different C sources [39]. The presence of preferred C sources (glucose is normally the preferred C for the bacteria [39]) prevents the expression, and often also the activity, of catabolic systems that enable the use of secondary substrates [39]. This result may also support the changes of bacterial communities in the presence of AMF hyphae [40]. AMF release different sugars and carboxylates at different concentrations into the soil that will stimulate certain groups of bacteria having preferences for some sugars more than others. This results in a quantitative modification in the bacterial populations and thus in the change of community composition of bacteria in contact with the hyphae.

Within the short-term period of study (i.e., 4 weeks), we expected the sugars to be exuded principally by the extraradical hyphae and to a lesser extent via cell death of hyphae. According to the reciprocity theory, the sugars released by AMF may stimulate bacterial activity and thus the breakdown of organic compounds into inorganic nutrients directly available to the hyphae. Here, we hypothesized that the sugars released by the AMF and taken up by the bacteria may stimulate bacterial cell division (by providing C source) and phosphatase activities (by providing signal molecules). However, the expression of two key genes in cell division (*ftsA* and *ftsZ*) was not stimulated by the presence of hyphae within the time frame of the experiment (from 1 to 72 h), while within a period of 2 and 4 weeks growth, the expression of these genes was stimulated [15], suggesting that AMF did not visibly influence bacterial growth in the short term. Stimulation of the expression of phosphatase genes can also enhance phosphatase activity [41]. In our study, the expression of four phosphatase genes was significantly stimulated by the hyphae of the AMF. It supported our second hypothesis that the expression of genes encoding bacterial phosphatase production and exudation increased concomitantly with the uptake of specific sugars. Interestingly, the expression of *fruT* followed the same trend as *acp1* and *alp*: they increased significantly in bacteria at 1 h and then decreased at 6 h. It seemed that the uptake of fructose may be responsible for the increase of *acp1* and *alp* expression, but the influence of other metabolites in the hyphal exudates could not be excluded. We then set different concentrations of fructose added to *R. aquatilis* in the Petri plates to test the effects of fructose uptake on the gene expression of *acp1* and *alp*. We noticed that the presence of fructose at a concentration ranging from 20 μM (the approximate concentration detected in the hyphal exudates) to 1 mM induced a greater expression of *fruT*, *acp1*, and *alp* and the expression of *fruT* had a positive linear correlation with the expression of *acp1* and *alp*. Conversely, the presence of glucose at the concentration of 20 μM (the approximate concentration detected in the hyphal exudates) did not increase the gene expression of *acp1* and *alp*. These results clearly demonstrate that the uptake of fructose by *R. aquatilis* triggered the expression of phosphatase genes. After being transferred across the membrane of *R. aquatilis*, fructose was phosphorylated to fructose 1-phosphate [42], which may serve as a signal molecule to stimulate the expression of other genes. Previous studies have shown that fructose-1-phosphate is the preferred effector of the catabolite repressor/activator, which can stimulate the expression of other genes in the soil bacterium *Pseudomonas putida* [42]. In our study, the fructose secreted by the fungus triggered the gene expression of phosphatases of *R. aquatilis* and thus increased the phosphatase activity in the hyphosphere. Besides fructose, trehalose has been demonstrated to play roles as signal molecule [13] and glucose is normally a preferred substrate for bacterial growth [43], but there is no evidence that they could also stimulate the gene expression of phosphatases. Besides sugars, there are also other C-rich compounds, e.g., carboxylates, amino acids in the hyphal exudates [23], but whether they can play roles as signal molecules needs further investigation.

Only the phosphatases released from bacterial cells are involved in organic P hydrolyzation. We selected five secreted phosphatases whose exudation usually depends on type II and IV secretory systems [44, 45]. We tested the expression of two genes, i.e., *gspF* in type II and *vib8* in type IV secretory systems and found that they were stimulated by AMF at 12 h and 6 to 48 h, respectively, which followed the increased expression of phosphatase genes. This suggested that AMF could increase the rate of phosphatase secretion by regulating the protein secretory systems in the bacterium.

The stimulated expression of phosphatase and secretory system genes may lead to higher phosphatase activity outside the bacterial cells. This was demonstrated by the significantly greater acid and alkaline phosphatase activities in the MSR medium sampled at 72 h, which was in agreement with our previous result at 2 and 4 week [15]. The increased phosphatase activities then enhanced phytate P hydrolyzation. The concentration of phytate P decreased by 120 μM in the presence of the AMF, and the decrease due to *R. aquatilis* immobilization was only 15 μM, which meant that most of the mobilized inorganic P was taken up by the AMF hyphae. The increased P uptake may be accompanied with more high-affinity P transporters in the extraradical hyphae. This was demonstrated by the significantly higher *GintPT* expression when AMF was present in the whole plant system. In addition, the expression of polyP synthesis gene *Vtc4p* was also increased, which indicated that the polyP synthesis process in the vacuoles was stimulated. These results clearly supported that AMF acquired their available P by stimulating the activity of PSB and supported our third hypothesis that the genes involved in the processes of phosphate transfer and polyP synthesis in the extraradical hyphae of the AMF was enhanced by the presence of the bacteria.

## Conclusion

**Fig. 8:**
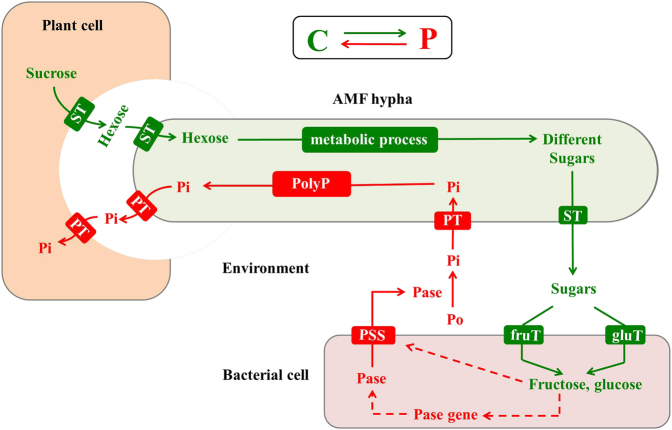
Schematic representation of the reciprocal rewards of carbon (C) and phosphorus (P) between the arbuscular mycorrhizal fungus (AMF) *R. irregularis* and the phosphate-solubilizing bacterium (PSB) *R. aquatilis*. ST sugar transporter, fruT fructose transporter, gluT glucose transporter, PT phosphate transporter, PSS protein secretory system, Pase phosphatase, Pi inorganic P, Po organic P

Our results demonstrated for the first time the reciprocal rewards of C and P between the AMF *R. irregularis* and the PSB *R. aquatilis*, at transcriptional level (Fig. 8). The extraradical hyphae of *R. irregularis* released sugars into the environment. The sugars were taken up by *R. aquatilis* in different orders of preference: fructose first and then glucose. Fructose exuded by the fungus played a key role as signal compound and stimulated the expression of phosphatase genes in the bacterium. AMF also enhanced the efficiency of phosphatase secretion to the environment by regulating the protein secretory systems in the bacterium. As a result, the phosphatase activity in the environment was increased and promoted the hydrolyzation of organic P. More inorganic P was thus available for the AMF and the processes relative to P uptake and metabolism were stimulated. Considering the ubiquity of AMF and their associated microbes [9, 46], our finding could help explaining the complex processes occurring in the rhizosphere that ultimately contribute to one of the acknowledged beneficial effects of arbuscular fungi to their host plants: the provision of soil phosphorus.

## Electronic supplementary material

Supplementary Information
