# Supplementary material for: Signal beyond nutrient, fructose, exuded by an arbuscular mycorrhizal fungus triggers phytate mineralization by a phosphate solubilizing bacterium
Source: ISME J. 2018 Jun 13;12(10):2339–51. doi: 10.1038/s41396-018-0171-4 (PMC6155042; doi:10.1038/s41396-018-0171-4)
Supplement: Supplementary file 1 — Supplementary Information [file 41396_2018_171_MOESM1_ESM.docx]

**Supplementary Information**

**Methods**

**Experiment 1: Fructose, glucose and trehalose exudation by hyphae of *R. irregularis***

*Carrot roots in vitro culture details*

Bi-compartmented Petri plates (90 × 15 mm) were used to grow the excised transformed carrot roots and AMF as detailed in St-Arnaud *et al*. (1996). Briefly, in the first compartment (i.e. the root compartment – RC), 25 ml MSR medium containing 3 g L^-1^ Phytagel was poured. In the second compartment (i.e. the hyphal compartment – HC), 4 ml MSR medium containing 3 g L^-1^ Phytagel without sucrose and vitamins was added in a slope from top to bottom of the plastic barrier in the HC (Zhang *et al*., 2016). Five mm of MSR medium was removed with sterilized scalpel along the plastic barrier in the RC to avoid diffusion of sugars from RC to HC (see Fig. S1). An excised transformed root of carrot associated with *R. irregularis* was transferred in the RC and incubated in a growth chamber at 27°C in the dark. After five weeks, a profuse extraradical mycelium (ERM) was observed and crossed the plastic barrier to grow in the slope. Two additional control treatments were included under strictly identical conditions: (1) Petri plates without roots in the RC and (2) Petri plates with AMF-colonized carrot roots grown in the RC without proliferation of ERM in the HC.

*Determination of sugars by ICS-3000 Ion Chromatography System*

The MSR medium was then collected, passed through an Acrodisc^®^ Syringe Filter and stored at -20°C until analysis. The hyphae remaining in the HC were cleaned three times with sterilized deionized water, collected and weighed. Hyphae were subsequently frozen at -80°C and ground in a Lysing Matrix tube (MP Biomedicals, Santa Ana, CA, USA) and the content extracted with 1.5 ml solution of methanol, chloroform and deionized water in proportion 5:2:2 by ultrasonic wave for two times during 30 min. The solution was then centrifuged for 10 min at the speed of 11000 rpm and 1 ml of the supernatant was extracted and vortexed with 0.6 ml deionized water and 0.3 ml chloroform. The solution was again centrifuged for 5 min at the speed of 4000 rpm, and the supernatant extracted ([Duan *et al.*, 2015](#_ENREF_19)). The presence and concentration of fructose, glucose and trehalose in the hyphal extract and the collected medium was determined by their respective standard sample (J&K Scientific Ltd., Beijing, China) using ICS-3000 Ion Chromatography System (Dionex, California, USA) ([Chi *et al.*, 2011](#_ENREF_11)). For the analysis in hyphal exudate, the RC^−MR^/HC^−RI^ treatment was used as the control, the RC^+MR^/HC^−RI^ treatment to test whether there is an influence of volatile compounds produced by roots and mycorrhizal hyphae and the RC^+MR^/HC^+RI^ treatment to collect the hyphal exudates.

**Experiment 5: Influence of *R. aquatilis* on gene expression in the extraradical hyphae of *R. irregularis***

*Autotrophic in vitro culture system details*

An autotrophic *in vitro* culture system was used (adapted from Voets *et al*. (2005)). Briefly, bi-compartment Petri plates were used to physically separate a RC from a HC. A small opening was made at the side of the RC. Twenty ml of MSR medium without sucrose and vitamins and solidified with 3 g L^-1^ Phytagel was poured in the RC and 4 ml in a slope from top to bottom of the plastic barrier in the HC.

Seven-day-old *M. truncatula* seedlings were transferred to the RC, with the roots on the surface of the medium and the shoot extending outside the system. In half of the systems, the plantlets were inoculated with ± 100 spores of *R. irregularis*. The other half was not inoculated. The systems were then sealed with Parafilm (Pechiney, Plastic Packaging, Chicago, IL 60631, USA) and the openings plastered with sterilized (121°C for 15 min) silicon grease (VWR International, Belgium). The systems were subsequently wrapped with opaque plastic bags to keep the AMF and plant roots in the dark, while shoots developed under light conditions. The systems were transferred to a growth chamber at 22/18°C (day/night), 70% relative humidity, photoperiod of 16 h day^-1^ and an average photosynthetic photon flux of 225 µmol m^-2^ s^-1^.

**References**

1. St-Arnaud M, Hamel C, Vimard B, Caron M, Fortin JA. Enhanced hyphal growth and spore production of the arbuscular mycorrhizal fungus *Glomus intraradices* in an *in vitro* system in the absence of host roots. Mycol Res. 1996; 100: 328-332.

2. Zhang L, Jiang C, Zhou J, Declerck S, Tian C, Feng G. Increasing phosphorus concentration in the extraradical hyphae of *Rhizophagus irregularis* DAOM 197198 leads to a concomitant increase in metal minerals. Mycorrhiza. 2016; 26: 909-918.

3. Duan L, Qi X, Chen M, Huang L. Application of metabolomics in the identification of Chinese herbal medicine. In Qi X, Chen X, Wang Y (eds). Plant Metabolomics: Methods and Applications. Chemical Industry Press: Beijing; Springer, Dordrecht. 2015; pp 227-244.

4. Chi Z, Zheng Y, Jiang A, Chen S. Lipid production by culturing oleaginous yeast and algae with food waste and municipal wastewater in an integrated process. Appl Biochem Biotechnol. 2011; 165: 442-453.

5. Voets L, Dupre de Boulois H, Renard L, Strullu DG, Declerck S. Development of an autotrophic culture system for the *in vitro* mycorrhization of potato plantlets. FEMS Microbiol Lett. 2005; 248: 111-118.

**Table S1** Summary of primers of *R. aquatilis* used in this study.

| Gene | Abbreviations  in this study | GenBank  accession number | Gene  length (bp) | Primer sequence  (5’-3’) | Product  length (bp) | PCR  efficiency |
| --- | --- | --- | --- | --- | --- | --- |
| PTS system fructose-specific transporter subunit IIA/HPr protein | *fruT* | AFE57532 | 1131 | AGGACGCTATTCGCCAGGTT  CCGTTGCCAAGGTAAGTAGAGGT | 115 | 0.95 |
| PTS system glucose-like transporter subunit IIB | *gluT* | AFE56875 | 1554 | GCCGATGCTGTATGTCCTG  GAAAGTCTGTCCGATGGTGA | 88 | 0.93 |
| PTS system trehalose (maltose)-specific transporter subunit IIBC | *treT* | AFE56783 | 1419 | TTGGCGAAGCCGTGTTTA  TTGCTGCCCGAGGTTGTA | 146 | * |
| glucose-1-phosphatase/inositol phosphatase | *phy* | AFE59656 | 1302 | GGCATGACAAGAAAGCGGATAAGG  AAATTGTTCCATCGGGCAGAAGC | 173 | 0.99 |
| acid phosphatase | *acp1* | AFE56808 | 699 | GGTGGACGATCACGCCAAGAAAG  TCGCCATCAGATAGCCGATAGTAGAA | 137 | 0.86 |
| acid phosphatase (plasmid) | *acp2* | AFE60737 | 738 | GCGACCACCAAACCAGACC  GCAGCAGACGACCCTGTTCATAC | 139 | 0.97 |
| acid phosphatase/phosphotransferase | *acp3* | AFE59341 | 714 | GCGGCGACAGCATTTACTTC  TTTGTCCTGCGGGATTTGG | 98 | 0.76 |
| alkaline phosphatase | *alp* | AFE58225 | 1431 | GCGTGGATGTAAACGGTAAA  CGTAGCACTTGCGTGAGG | 147 | 0.85 |
| cell division protein FtsA | *ftsA* | AFE60005 | 1257 | GATGGCGGATTGCCAAAT  CTGCGTGACTTCCTCTTCTG | 109 | 1.09 |
| cell division protein FtsZ | *ftsZ* | AFE60004 | 1155 | ACCGTTGCTGTTGTGACTAAG  GCTTGTCGTTCGGGATAG | 124 | 0.91 |
| type II secretion system protein GspF | *gspF* | AFE56600 | 1218 | TGCCATTTCACTATTCTCAC  TCCACCACTTTGTTACGC | 144 | 0.93 |
| type IV secretion system protein VirB8 | *vib8* | AFE58139 | 684 | CTCACTTACCGTTATGTCCC  ATTCGCTCCTCAGTTCTTT | 112 | 0.84 |
| RNA polymerase sigma factor RpoD | *rpoD* | AFE56820 | 1839 | ACTTGTCACCCGTGGTAAGGAGC  TTCGTCGGTATCAGGGCGGTTTT | 196 | 0.96 |

* PCR efficiency is not calculated due to the low express of the gene in the serial of diluted cDNA samples.

**Table S2** ANOVA output results of experiment 2.

(a) One-way ANOVA output of the repeated-measures analysis for acid phosphatase (ACP) activity, alkaline phosphatase (ALP), inorganic P concentration and phytate-P decreased concentration in the liquid MSR medium harvested from the hyphal compartment of the Petri plates in experiment 2.

| Source of variation |  | AMF | | |
| --- | --- | --- | --- | --- |
|  |  | d.f. | F | P |
| Phosphatase activity |  |  |  |  |
| ACP |  | 2, 9 | 237.3 | *** |
| ALP |  | 2, 9 | 781.7 | *** |
| P concentration |  |  |  |  |
| Inorganic P |  | 2, 9 | 32.0 | *** |
| Phytate-P decreased |  | 2, 9 | 14.2 | ** |

Asterisks indicate significance levels. ***P* < 0.01; ****P* < 0.001.

(b) Two-way ANOVA output of the repeated-measures analysis for the expression of putative sugar transporter genes (*fruT* and *gluT*), phosphatase genes (*phy*, *acp1*, *acp2*, *acp3* and *alp*), key genes in cell division (*ftsA* and *ftsZ*) and genes involved in secretion systems (*gspF* and *vib8*) of *R. aquatilis* harvested from the hyphal compartment of the Petri plates in experiment 2.

| Source of variation |  | Time | | |  | AMF | | |  | Time × AMF | | |
| --- | --- | --- | --- | --- | --- | --- | --- | --- | --- | --- | --- | --- |
|  |  | d.f. | F | P |  | d.f. | F | P |  | d.f. | F | P |
| Putative sugar transporter |  |  |  |  |  |  |  |  |  |  |  |  |
| *fruT* |  | 5, 54 | 52.6 | *** |  | 2, 54 | 2.3 | n.s. |  | 10, 54 | 29.8 | *** |
| *gluT* |  | 5, 54 | 90.0 | *** |  | 2, 54 | 296.3 | *** |  | 10, 54 | 34.4 | *** |
| Phosphatase |  |  |  |  |  |  |  |  |  |  |  |  |
| *phy* |  | 5, 54 | 16.5 | *** |  | 2, 54 | 99.6 | *** |  | 10, 54 | 2.9 | ** |
| *acp1* |  | 5, 54 | 429.0 | *** |  | 2, 54 | 338.7 | *** |  | 10, 54 | 339.8 | *** |
| *acp2* |  | 5, 54 | 47.7 | *** |  | 2, 54 | 88.7 | *** |  | 10, 54 | 16.8 | *** |
| *acp3* |  | 5, 54 | 5.7 | *** |  | 2, 54 | 31.1 | *** |  | 10, 54 | 5.5 | *** |
| *alp* |  | 5, 54 | 68.1 | *** |  | 2, 54 | 63.6 | *** |  | 10, 54 | 58.9 | *** |
| Cell division |  |  |  |  |  |  |  |  |  |  |  |  |
| *ftsA* |  | 5, 54 | 5.6 | *** |  | 2, 54 | 6.9 | ** |  | 10, 54 | 1.9 | n.s. |
| *ftsZ* |  | 5, 54 | 4.3 | ** |  | 2, 54 | 0.2 | n.s. |  | 10, 54 | 1.5 | n.s. |
| Secretion system |  |  |  |  |  |  |  |  |  |  |  |  |
| *gspF* |  | 5, 54 | 7.6 | *** |  | 2, 54 | 10.7 | *** |  | 10, 54 | 8.1 | *** |
| *vib8* |  | 5, 54 | 21.3 | *** |  | 2, 54 | 88.1 | *** |  | 10, 54 | 11.1 | *** |

Asterisks indicate significance levels. n.s., *P* ≥ 0.05; ***P* < 0.01; ****P* < 0.001.

**Fig. S1** Schematic representation of experiment 1. Five mm MSR medium was removed along the plastic barrier in the root compartment. Red lines, extraradical hyphae of *R. irregularis*. Treatment codes are as follows: RC^−MR^/HC^−RI^, absence of mycorrhizal roots in the RC and *R. irregularis* in the HC; RC^+MR^/HC^−RI^, presence of mycorrhizal roots in the RC but without proliferation of *R. irregularis* in the HC; RC^+MR^/HC^+RI^, presence of mycorrhizal roots in the RC and proliferation of *R. irregularis* in the HC. RC, root compartment; HC, hyphal compartment; RI, *R. irregularis*.

**Fig. S2** Schematic representation of experiment 2. Red lines, extraradical hyphae of *R. irregularis*; green dots, bacterial cells of *R. aquatilis*. Treatment codes are as follows: RC^−MR^/HC^−RI^, absence of mycorrhizal roots in the RC and *R. irregularis* in the HC; RC^+MR^/HC^−RI^, presence of mycorrhizal roots in the RC but without proliferation of *R. irregularis* in the HC; RC^+MR^/HC^+RI^, presence of mycorrhizal roots in the RC and proliferation of *R. irregularis* in the HC. RC, root compartment; HC, hyphal compartment; RA, *R. aquatilis*; RI, *R. irregularis*.

**Fig. S3** Schematic representation of experiment 5. Red lines, extraradical hyphae of *R. irregularis*; green dots, bacterial cells of *R. aquatilis*. Treatment codes are as follows: –RI–RA, absence of *R. irregularis* and *R. aquatilis* in the HC; –RI+RA, absence of *R. irregularis* but with *R. aquatilis* in the HC; +RI–RA, presence of *R. irregularis* but without *R. aquatilis* in the HC; +RI+RA, presence of both *R. irregularis* and *R. aquatilis* in the HC. HC, hyphal compartment; RA, *R. aquatilis*; RI, *R. irregularis*.
